# Identification of Plasmid-Mediated Tigecycline-Resistant Gene *tet*(X4) in *Enterobacter cloacae* from Pigs in China

**DOI:** 10.1128/spectrum.02064-21

**Published:** 2022-03-01

**Authors:** Yiping Wu, Ruowen He, Mingyang Qin, Yanxian Yang, Jieyun Chen, Yu Feng, Xiaoxue Liang, Wenbin Deng, Xin Ding, Li-Na Qin, Kang Liao, Yongqiang Yang, Guo-Bao Tian

**Affiliations:** a Department of Microbiology, Zhongshan School of Medicine, Sun Yat-sen University, Guangzhou, China; b Key Laboratory of Tropical Diseases Control, Sun Yat-sen University, Ministry of Education, Guangzhou, China; c School of Basic Medical Sciences, Xinxiang Medical University, Xinxiang, China; d School of Laboratory Medicine, Chengdu Medical College, Chengdu, China; e School of Pharmaceutical Sciences (Shenzhen), Sun Yat-sen University, Guangzhou, China; f Department of Histology and Embryology, Zhongshan School of Medicine, Sun Yat-sen University, Guangzhou, China; g Department of Clinical Laboratory, the First Affiliated Hospital of Sun Yat-Sen University, Guangzhou, China; h School of Medicine, Xizang Minzu University, Xianyang, Shaanxi, China; Forschungszentrum Jülich GmbH

**Keywords:** *tet*(X4), tigecycline resistance, *Enterobacter cloacae*

## Abstract

Two *tet*(X4)-positive Enterobacter cloacae isolates TECL_1 and TECL_2 were isolated from pigs in China. S1-PFGE and Southern blotting showed that *tet*(X4) located on plasmids in the size of ∼290 kb and ∼190 kb in TECL_1 and TECL_2, respectively. Conjugation experiment demonstrated that the *tet*(X4)-harboring plasmid can transfer from the donor strain TECL_1 and TECL_2 to the recipient strain Escherichia coli J53, and the tigecycline resistance of transconjugants was increased by 128-fold and 64-fold compared with E. coli J53, respectively. We obtained the complete plasmid sequence of pTECL_2-190k-tetX4 (190,185 bp) from E. cloacae TECL_2 and found that the plasmid was a hybrid plasmid with replicon types of IncFIA, IncHI1A and IncHI1B. We further analyzed 85 *tet*(X4)-carrying plasmids in the public database and clarified that pTECL_2-190k-tetX4-like plasmid was widespread in multiple species of Enterobacteriaceae.

**IMPORTANCE** We identified two *tet*(X4)-positive E. cloacae isolates, which has not been previously reported. We obtained the complete sequence of pTECL_2-190k-tetX4 and found that it was a hybrid plasmid with multiple replicon types, including IncFIA, IncHI1A and IncHI1B. By comparing all the known *tet*(X4)-carrying plasmids, we found that pTECL_2-190k-tetX4-like plasmid has been disseminated across various species in China. Our study expanded the identification of *tet*(X4)-positive species and emphasized that pTECL_2-190k-tetX4-like plasmid has spread widely in various species.

## OBSERVATION

Enterobacter cloacae is one of the members of Enterobacteriaceae, which was reported as an important opportunistic microbial pathogen for a broad range of hospital-acquired infections (1). Tigecycline is a last-resort antibiotic for the treatment of life-threatening infections caused by multidrug-resistant bacteria, such as carbapenem-resistant Enterobacteriaceae (2). In recent years, the tigecycline resistance gene *tet*(X) has been reported to mediate high-level resistance to all tetracycline antibiotics in isolates from animals, humans and the environment, posing a significant risk to public health (3–5). In 2019, a plasmid-borne high-level tigecycline resistance gene *tet*(X) was identified (6). Herein, we identified two E. cloacae isolates harboring plasmid-mediated *tet*(X) gene, which has not been reported, and further analyzed the genetic context of the *tet*(X4)-carrying plasmid pTECL_2-190k-tetX4.

We collected 590 nonduplicate samples, including 475 pig nasal swabs, 67 pig anal swabs and 48 staff skin swabs from a pig farm and a slaughterhouse in Guangdong Province. Then colonies were selected from BHI plate containing 4 mg/L tigecycline after preinoculation and screened for *tet*(X4) variants by PCR (7). Finally, we identified two E. cloacae strains TECL_1 and TECL_2 carrying *tet*(X4). MICs of 14 antimicrobial agents for strains were determined (Table S1). Both strains were resistant to tigecycline, tetracycline, rifamycin, ampicillin, chloramphenicol and ciprofloxacin. In addition, TECL_1 was resistant to fosfomycin; TECL_2 was resistant to ceftazidime, colistin sulfate, cefotaxime, gentamicin, and trimethoprim-sulfamethoxazole. Then the two E. cloacae isolates were subjected to genomic DNA extraction and whole-genome sequencing. The sequencing reads were assembled into contigs using SPAdes version 3.10 (8). Antibiotic resistance genes (ARGs) were predicted using ResFinder v3.2 (9). It showed that TECL_1 harbored nine ARGs including *tet*(X4), *tet*(M), *aadA2*, *aadA22*, *bla*_TEM-1B_, *qnrS1*, *lnu*(G), *lnu*(F) and *floR*. TECL_2 carried 21 ARGs including *tet*(X4), *tet*(A), *ant(3'')-Ia*, *aadA16*, *aac(6')-Ib-cr*, *aac(3)-IId*, *aph(6)-Id*, *aph(3')-Ia*, *aph(3'')-Ib*, *bla*_CTX-M_, *bla*_CMH-3_, *oqxA*, *oqxB*, *qnrS1*, *lnu*(G), *fosA*, *mph*(A), *sul1*, *sul2*, *arr-3* and *dfrA27*.

**FIG 1:**
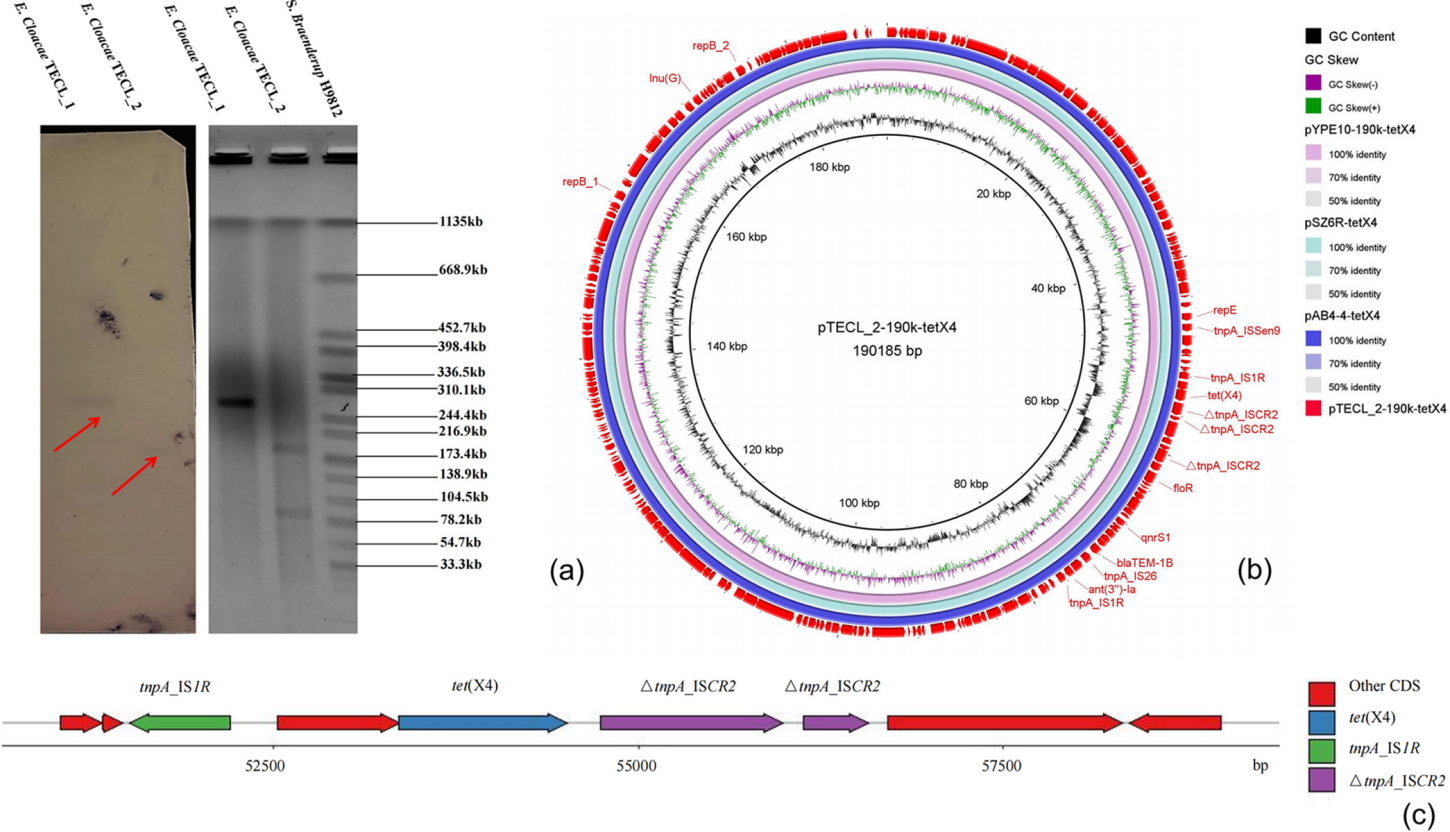
The plasmid structure of pTECL_2-190k-tetX4. (a) The location of *tet*(X4) in E. cloacae isolates TECL_1 and TECL_2 by S1-PFGE and Southern blotting. Salmonella *Braenderup* strain H9812 was used as the marker by *Xba*I enzyme digestion. (b) The circular genetic map of pTECL_2-190k-tetX4, pYPE10-190k-tetX4 (GenBank accession No. CP041449.1), pSZ6R-tetX4 (GenBank accession No. MW940627.1) and pAB4-4-tetX4 (GenBank accession No. MW940615.1). The arrows on the outer circle represent the genes of replicon, antibiotic resistance, and transposase. The three middle circles show the similarity of three plasmids harboring *tet*(X4) with pTECL_2-190k-tetX4. The inner arc represents GC skew curve and the next represents GC contents. (c) Major structural features of the *tet*(X4) gene. The blue arrow represents *tet*(X4). The green arrow represents the transposase of the IS*1R* element and the transposase of the ΔIS*CR2* element is purple. The number marked on the ruler at the bottom of the picture corresponds to the nucleotide position on the plasmid.

Previous studies reported that *tet*(X4) is mostly located on the plasmid in Enterobacteriaceae (10, 11). To determine the transferability of *tet*(X4)-harboring plasmids in E. cloacae isolates, we performed the conjugation experiment. It showed that *tet*(X4) could be successfully transferred from TECL_1 and TECL_2 into the recipient E. coli J53 by filter mating. The transconjugants J53/pTECL_1-290k-tetX4 and J53/pTECL_2-190k-tetX4 were resistant to tigecycline with MIC values of 32 and 16 mg/L, respectively. The tigecycline resistance of two transconjugants was increased by 128-fold and 64-fold compared with E. coli J53 (Table S1). Then PCR and Sanger sequencing demonstrated that the transconjugants carried *tet*(X4). Furthermore, S1-PFGE and Southern blotting hybridization revealed that *tet*(X4) located on plasmids in the size of ∼290 kb and ∼190 kb in TECL_1 and TECL_2, respectively (Fig. 1a). The results proved that *tet*(X4) was located on the plasmid of E. cloacae isolates, and could be transmitted to other species, causing the spread of tigecycline resistance in Enterobacteriaceae.

To determine the complete sequences of pTECL_2-190k-tetX4, we combined the sequencing data from the genomic DNA and the plasmids and closed predicted gaps within the sequences by PCR and Sanger sequencing using primers listed in Table S2. Finally, we obtained the complete sequence of pTECL_2-190k-tetX4 from E. cloacae strain TECL_2. The sequence was analyzed using the method mentioned in the previous article (12). pTECL_2-190k-tetX4 was a 190,185 bp plasmid with three replicon types IncFIA, IncHI1A and IncHI1B, and encoded 220 predicted ORFs (Fig. 1b). pTECL_2-190k-tetX4 showed a mosaic structure harboring six ARGs, including *tet*(X4) along with *ant*(3')-*Ia*, *bla*_TEM-1B_, *lnu*(G), *floR* and *qnrS1*. The *tet*(X4) gene, was flanked by a complete IS*1R* element and a truncated IS*CR2* element. This IS*1R* element was located at 1099 bp upstream of *tet*(X4). The downstream region of *tet*(X4) was a 223 bp fragment encoding the transposase of IS*CR2* element. There is a 136 bp fragment insertion resulting in the truncation of IS*CR2* element. (Fig. 1c). BLASTn of pTECL_2-190k-tetX4 against the nr database retrieved similar plasmids from different hosts. The plasmid pTECL_2-190-tetX4 was highly similar with pYPE10-190k-tetX4 isolated from E. coli strain YPE10 (CP041449.1, 99.96% identity at 100% coverage) (13), pSZ6R-tetX4 isolated from Citrobacter braakii strain SZ6R (MW940627.1, 99.92% identity at 100% coverage) (14) and pAB4-4-tetX4 isolated from Klebsiella pneumoniae strain AB4-4 (MW940615.1, 99.92% identity at 100% coverage) (14). The result indicated that the pTECL_2-190k-tetX4-like plasmid might have been widely spread in different species of Enterobacteriaceae.

**FIG 2:**
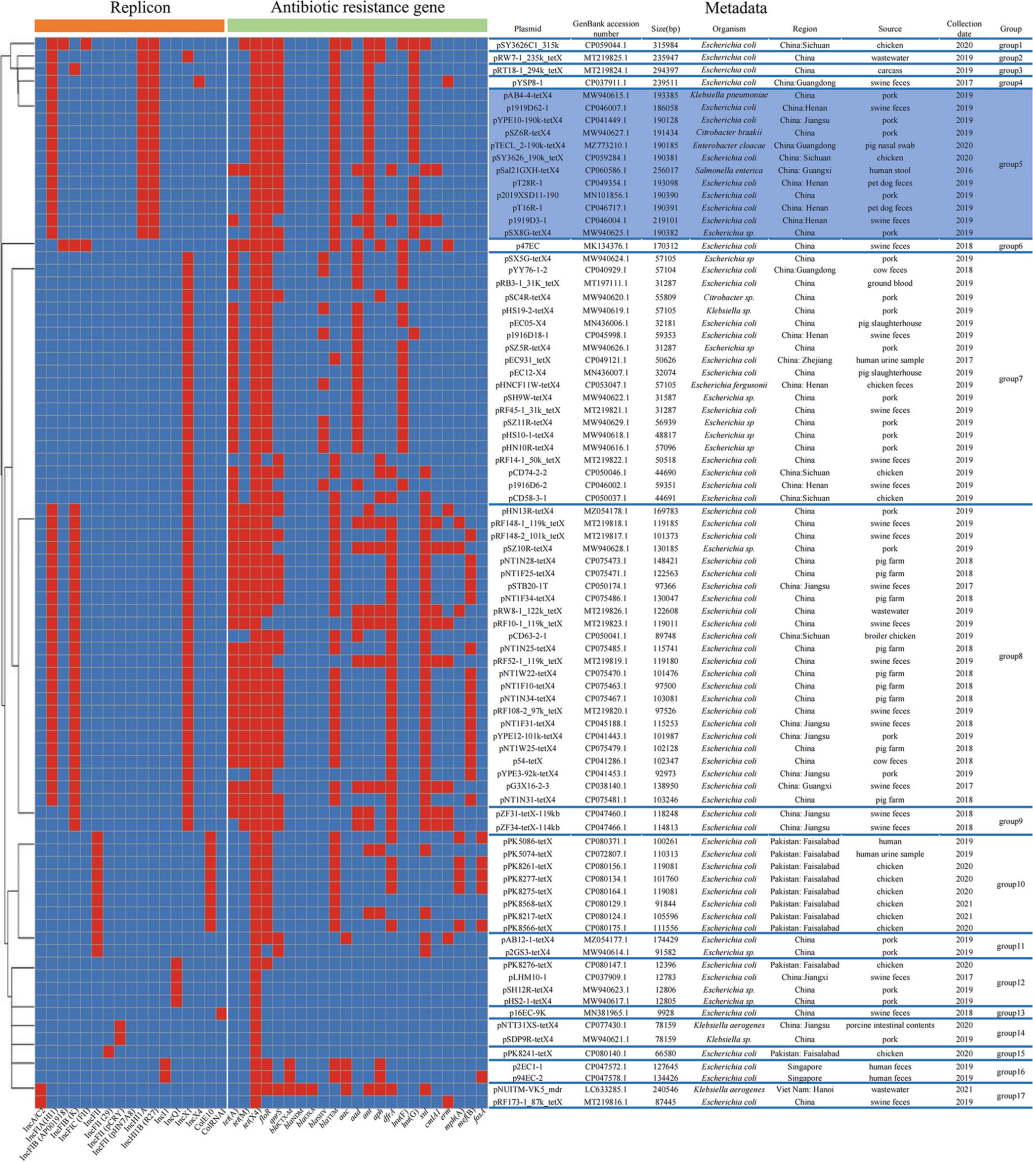
The comparison of the publicly available plasmids carrying *tet*(X4). The average-linkage clustering method was used to cluster 85 plasmids carrying *tet*(X4) according to the replicon type. The groups were separated by blue horizontal lines in the figure. The prominent part of the blue block is the group which pTECL_2-190k-tetX4 belongs. The distribution of all replicon types and antibiotic resistance genes were presented by heatmap. In terms of whether the corresponding plasmid replicon and antibiotic resistance genes is present in the plasmid, red represents presence and blue represents absence. The metadata of plasmids is shown on the right of figure, including the name, GenBank accession number, size, and source of plasmids.

It was reported that the plasmid carrying *tet*(X4) showed structure diversity (15). We compared all the known *tet*(X4)-carrying plasmids and tried to clarify the prevalence of plasmids carrying *tet*(X4), especially the pTECL_2-190k-tetX4-like plasmid. BLASTn of the *tet*(X4) gene identified 90 publicly available *tet*(X4)-carrying plasmids, as of 7 August 2021. We excluded three transconjugative plasmids, one plasmid with the sequence of repeated uploads and two plasmids without replicon sequence. The remaining 84 complete plasmid sequences were compared with pTECL_2-190k-tetX4 (Fig. 2). The 85 plasmids carrying *tet*(X4) were clustered 17 types of plasmid group by the different replicon types. pTECL_2-190k-tetX4-like plasmids (at least 99.91% identity at 92% coverage with pTECL_2-190k-tetX4) are one of the dominant type of plasmids harboring *tet*(X4) (*n* = 12). The size of the plasmids is about 200 kb. Besides *tet*(X4), multiple ARGs were co-existed in these plasmids such as *floR*, *qnrS1*, *bla*_TEM-1B_, *ant(3'')-Ia* and *lnu*(F) (Table S3). Strains carrying pTECL_2-190k-tetX4-like plasmid have been found from swine, pet dog and chicken in China, indicating that this type of plasmid has disseminated widely in animals in China (Fig. 2). E. cloacae TECL_2 was isolated from a pig farm in Guangdong Province, indicating the transmission range of the plasmid in China has been further expanded. In addition, it is noteworthy that the host bacteria of this plasmid are diverse, such as E. coli, E. cloacae, S. enterica, K. pneumoniae and *C. braa*kii, indicating this plasmid has strong host adaptability.

In conclusion, the identification of two *tet*(X4)-positive E. cloacae isolates indicates that the host range of *tet*(X4) has been further expanded. In addition, the widespread of pTECL_2-190k-tetX4-like plasmid in Enterobacteriaceae must be concerned.

### Data availability.

This complete sequence for pTECL_2-190k-tetX4 has been deposited in GenBank under the accession number MZ773210.1.
