# Supplementary material for: Identification of Plasmid-Mediated Tigecycline-Resistant Gene tet(X4) in Enterobacter cloacae from Pigs in China
Source: Microbiol Spectr. 2022 Mar 1;10(2):e02064-21. doi: 10.1128/spectrum.02064-21 (PMC9045145; doi:10.1128/spectrum.02064-21)
Supplement: SUPPLEMENTAL FILE 1 — Supplemental material. Download SPECTRUM02064-21_Supp_1_seq7.pdf, PDF file, 0.3 MB [file spectrum02064-21_supp_1_seq7.pdf]

TABLE S1 Antimicrobial susceptibility profiles of bacterial hosts carrying *tet*(X4)

Strains	*E. cloacae* TECL_1	*E. cloacae* TECL_2	*E. coli* J53/pTECL_1-290k-tetX4	*E. coli* J53/pTECL_2-190k-tetX4	*E. coli* J53
Description					
Source	Pig farm	Pig farm	/	/	/
Collection date	2020	2020	/	/	/
	Donor of *tet*(X4)-carrying plasmid pTECL_1-190k-tetX4	Donor of *tet*(X4)-carrying plasmid pTECL_2-290k-tetX4	Transconjugant	Transconjugant	Recipient
MICs(mg/L) [a]					
TET	256	256	128	128	≤1
TGC	32	16	32	32	0.25
RD	16	> 128	8	16	/
AMP	> 256	> 256	8	8	/
CHL	> 128	> 128	128	128	8
CIP	4	4	≤0.03	≤0.03	≤0.03
IMP	≤0.25	≤0.25	0.5	0.5	0.5
CAZ	2	32	≤1	≤1	≤1
GEN	≤1	256	≤1	≤1	≤1
AMK	≤1	2	2	2	≤1
CTX	≤1	256	≤1	≤1	≤1
FOS	256	≤16	≤16	≤16	/
CT	0.25	> 16	0.25	0.25	≤0.125
SXT	≤0.25	> 64	≤0.25	≤0.25	≤0.25

a. TET, tetracycline; TGC, tigecycline; RD, rifamycin; AMP, ampicillin; CHL, chloramphenicol; CIP, ciprofloxacin; IMP, imipenem; CAZ, ceftazidime; GEN, gentamicin; AMK, amikacin; CTX, cefotaxime; FOS, fosfomycin; CT, colistin sulphate; SXT, trimethoprim-sulfamethoxazole

TABLE S2 The primers for pTECL_2-190k-tetX4

Plasmid	Primer	Sequence (5' to 3')	Amplified product size（bp）	Position（bp）
pTECL_2-190k-tetX4	pTECL2-1-F	CGCCTTGCTGGTTGATGAAG	802	55191-56000
	pTECL2-1R	TGGAAGCGGGTTAGGTTGAC		
	pTECL2-2-F	GTGACGGGATTGGCAAGGTA	841	60459-61329
	pTECL2-2-R	ACATGGGCATCGACATAGCC		
	pTECL2-3-F	CTGCTATTTGAGCGCCATGC	582	63632-64215
	pTECL2-3-R	GTATCGAAGCGCTGAACGTC		

TABLE S3 The antibiotic resistance gene of plasmids carrying *tet*(X4)

Plasmid	GenBank accession number	Antibiotic resistance gene						
		Tetracyclines-resistance gene			Chloramphenicol-resistance gene		Quinolones-resistance gene	
		tet(A)	*tet*(M)	*tet*(X4)	*floR*	*cmlA1*	*qnrS1*	*qnrS2*
pTECL_2-190k-tetX4	MZ773210.1	0	0	1	1	0	1	0
pLHM10-1	CP037909.1	0	0	1	0	0	0	0
pYSP8-1	CP037911.1	0	0	1	1	0	1	0
pG3X16-2-3	CP038140.1	1	1	1	1	1	0	0
pYY76-1-2	CP040929.1	1	0	1	1	0	0	0
p54-tetX	CP041286.1	1	1	1	1	0	1	0
pYPE12-101k-tetX4	CP041443.1	1	1	1	1	0	1	0
pYPE10-190k-tetX4	CP041449.1	0	0	1	1	0	1	0
pYPE3-92k-tetX4	CP041453.1	0	0	1	1	0	0	0
pNT1F31-tetX4	CP045188.1	1	1	1	1	0	1	0
p1916D18-1	CP045998.1	1	0	1	1	0	0	0
p1916D6-2	CP046002.1	1	0	1	1	0	0	0
p1919D3-1	CP046004.1	1	0	1	1	1	0	0
p1919D62-1	CP046007.1	0	0	1	1	0	1	0
pT16R-1	CP046717.1	0	0	1	1	0	1	0
pZF31-tetX-119kb	CP047460.1	1	1	1	1	1	1	0
pZF34-tetX-114kb	CP047466.1	0	1	1	1	1	1	0
p2EC1-1	CP047572.1	0	0	1	1	0	0	0
p94EC-2	CP047578.1	0	0	1	1	0	0	0
pEC931_tetX	CP049121.1	1	0	1	1	0	0	0
pT28R-1	CP049354.1	0	0	1	1	0	1	0
pCD58-3-1	CP050037.1	1	0	1	1	0	1	0
pCD63-2-1	CP050041.1	0	0	1	1	0	1	0
pCD74-2-2	CP050046.1	1	0	1	1	0	1	0
pSTB20-1T	CP050174.1	1	1	1	1	0	1	0
pHNCF11W-tetX4	CP053047.1	1	0	1	1	0	0	0
pSY3626C1_315k	CP059044.1	0	1	1	1	0	1	0
pSY3626_190k_tetX	CP059284.1	0	0	1	1	0	1	0
pSal21GXH-tetX4	CP060586.1	1	1	1	1	1	1	0
pPK5074-tetX	CP072807.1	0	0	1	1	0	0	0
pNT1F10-tetX4	CP075463.1	1	1	1	1	0	1	0
pNT1N34-tetX4	CP075467.1	1	1	1	1	0	1	0
pNT1W22-tetX4	CP075470.1	1	1	1	1	0	1	0
pNT1F25-tetX4	CP075471.1	1	1	1	1	0	1	0
pNT1N28-tetX4	CP075473.1	1	1	1	1	0	1	0
pNT1W25-tetX4	CP075479.1	1	1	1	1	0	1	0
pNT1N31-tetX4	CP075481.1	1	1	1	1	0	1	0
pNT1N25-tetX4	CP075485.1	1	1	1	1	0	1	0
pNT1F34-tetX4	CP075486.1	1	1	1	1	0	1	0

pNTT31XS-tetX4	CP077430.1	0	0	1	0	0	0	0
pPK8217-tetX	CP080124.1	0	0	1	1	0	0	0
pPK8568-tetX	CP080129.1	0	0	1	1	0	0	0
pPK8277-tetX	CP080134.1	0	0	1	1	0	0	0
pPK8241-tetX	CP080140.1	0	0	1	0	0	0	0
pPK8276-tetX	CP080147.1	0	0	1	1	0	0	0
pPK8261-tetX	CP080156.1	0	0	1	1	0	0	0
pPK8275-tetX	CP080164.1	0	0	1	1	0	0	0
pPK8566-tetX	CP080175.1	0	0	1	1	0	0	0
pPK5086-tetX	CP080371.1	0	0	1	1	0	0	0
pNUITM-VK5_mdr	LC633285.1	0	0	1	1	0	1	0
p47EC	MK134376.1	1	1	1	1	0	1	0
p2019XSD11-190	MN101856.1	0	0	1	1	0	1	0
p16EC-9K	MN381965.1	0	0	1	0	0	0	0
pEC05-X4	MN436006.1	1	0	1	1	0	0	0
pEC12-X4	MN436007.1	1	0	1	1	0	0	0
pRB3-1_31K_tetX	MT197111.1	1	0	1	1	0	0	0
pRF173-1_87k_tetX	MT219816.1	0	0	1	0	0	0	0
pRF148-2_101k_tetX	MT219817.1	1	1	1	1	0	1	0
pRF148-1_119k_tetX	MT219818.1	1	1	1	1	1	0	0
pRF52-1_119k_tetX	MT219819.1	1	1	1	1	1	0	0
pRF108-2_97k_tetX	MT219820.1	1	1	1	1	0	1	0
pRF45-1_31k_tetX	MT219821.1	1	0	1	1	0	0	0
pRF14-1_50k_tetX	MT219822.1	0	0	1	0	0	1	0
pRF10-1_119k_tetX	MT219823.1	1	1	1	0	1	0	0
pRT18-1_294k_tetX	MT219824.1	0	0	1	1	0	1	0
pRW7-1_235k_tetX	MT219825.1	0	0	1	1	0	1	1
pRW8-1_122k_tetX	MT219826.1	1	1	1	1	1	0	0
p2GS3-tetX4	MW940614.1	0	0	1	0	0	1	0
pAB4-4-tetX4	MW940615.1	0	0	1	1	0	1	0
pHN10R-tetX4	MW940616.1	1	0	1	1	0	0	0
pHS2-1-tetX4	MW940617.1	0	0	1	0	0	0	0
pHS10-1-tetX4	MW940618.1	1	0	1	1	0	0	0
pHS19-2-tetX4	MW940619.1	1	0	1	1	0	0	0
pSC4R-tetX4	MW940620.1	0	0	1	1	0	0	1
pSDP9R-tetX4	MW940621.1	0	0	1	0	0	0	0
pSH9W-tetX4	MW940622.1	1	0	1	1	0	0	0
pSH12R-tetX4	MW940623.1	0	0	1	0	0	0	0
pSX5G-tetX4	MW940624.1	1	0	1	1	0	0	0
pSX8G-tetX4	MW940625.1	0	0	1	1	0	1	0
pSZ5R-tetX4	MW940626.1	1	0	1	1	0	0	0
pSZ6R-tetX4	MW940627.1	0	0	1	1	0	1	0
pSZ10R-tetX4	MW940628.1	1	1	1	1	1	0	0
pSZ11R-tetX4	MW940629.1	1	0	1	1	0	0	0

pAB12-1-tetX4	MZ054177.1	0	0	1	1	0	1	0
pHN13R-tetX4	MZ054178.1	1	1	1	1	0	1	0

Plasmid	Antibiotic resistance gene									
	β-lactams-resistance gene									
	*bla*CTX-M-14	*bla*CTX-M-65	*bla*NDM-4	*bla*OXA-9	*bla*SHV-12	*bla*TEM-135	*bla*TEM-176	*bla*TEM-1A	*bla*TEM-1B	*bla*TEM-215
pTECL_2-190k-tetX4	0	0	0	0	0	0	0	0	1	0
pLHM10-1	0	0	0	0	0	0	0	0	0	0
pYSP8-1	0	0	0	0	0	0	0	0	1	0
pG3X16-2-3	0	0	0	0	0	0	0	0	1	0
pYY76-1-2	0	0	0	0	1	0	0	0	0	0
p54-tetX	0	0	0	0	0	0	0	0	1	0
pYPE12-101k-tetX4	0	0	0	0	0	0	0	0	1	0
pYPE10-190k-tetX4	0	0	0	0	0	0	0	0	1	0
pYPE3-92k-tetX4	0	0	0	0	0	0	0	0	0	0
pNT1F31-tetX4	0	0	0	0	0	0	0	0	1	0
p1916D18-1	0	0	0	0	1	0	0	0	0	0
p1916D6-2	0	0	0	0	1	0	0	0	0	0
p1919D3-1	0	0	0	0	0	0	0	0	1	0
p1919D62-1	0	0	0	0	0	0	0	0	1	0
pT16R-1	0	0	0	0	0	0	0	0	1	0
pZF31-tetX-119kb	0	0	0	0	0	0	0	0	0	0
pZF34-tetX-114kb	0	0	0	0	0	0	0	0	0	0
p2EC1-1	0	1	0	0	0	0	0	0	1	0
p94EC-2	0	1	0	0	0	0	0	0	1	0
pEC931_tetX	0	0	0	0	0	0	0	0	1	0
pT28R-1	0	0	0	0	0	0	0	0	1	0
pCD58-3-1	0	0	0	0	0	0	0	0	1	0
pCD63-2-1	0	0	0	0	0	0	0	0	1	0
pCD74-2-2	0	0	0	0	0	0	0	0	1	0
pSTB20-1T	0	0	0	0	0	0	0	0	1	0
pHNCF11W-tetX4	0	0	0	0	1	0	0	0	0	0
pSY3626C1_315k	0	0	0	0	0	0	0	0	1	0
pSY3626_190k_tetX	0	0	0	0	0	0	0	0	1	0
pSal21GXH-tetX4	0	0	0	0	0	0	0	0	1	0
pPK5074-tetX	0	0	0	0	0	0	0	0	0	1
pNT1F10-tetX4	0	0	0	0	0	0	0	0	1	0
pNT1N34-tetX4	0	0	0	0	0	0	0	0	1	0

pNT1W22-tetX4	0	0	0	0	0	0	0	0	1	0
pNT1F25-tetX4	0	0	0	0	0	0	0	0	1	0
pNT1N28-tetX4	0	0	0	0	0	0	0	0	1	0
pNT1W25-tetX4	0	0	0	0	0	0	0	1	0	0
pNT1N31-tetX4	0	0	0	0	0	0	0	0	1	0
pNT1N25-tetX4	0	0	0	0	0	0	0	0	1	0
pNT1F34-tetX4	0	0	0	0	0	0	0	0	1	0
pNTT31XS-tetX4	0	0	0	0	0	0	0	0	0	0
pPK8217-tetX	0	0	0	0	0	0	0	0	0	1
pPK8568-tetX	0	0	0	0	0	1	0	0	0	0
pPK8277-tetX	0	0	0	0	0	0	0	0	0	1
pPK8241-tetX	0	0	0	0	0	0	0	0	0	0
pPK8276-tetX	0	0	0	0	0	0	0	0	0	0
pPK8261-tetX	0	0	0	0	0	0	0	0	0	1
pPK8275-tetX	0	0	0	0	0	0	0	0	0	1
pPK8566-tetX	0	0	0	0	0	0	0	0	0	1
pPK5086-tetX	0	0	0	0	0	0	0	0	0	1
pNUITM-VK5_mdr	1	0	1	1	0	0	0	0	0	0
p47EC	0	0	0	0	0	0	0	0	1	0
p2019XSD11-190	0	0	0	0	0	0	0	0	1	0
p16EC-9K	0	0	0	0	0	0	0	0	0	0
pEC05-X4	0	0	0	0	0	0	0	0	0	0
pEC12-X4	0	0	0	0	0	0	0	0	0	0
pRB3-1_31K_tetX	0	0	0	0	0	0	0	0	0	0
pRF173-1_87k_tetX	0	0	0	0	0	0	0	0	0	0
pRF148-2_101k_tetX	0	0	0	0	0	0	0	0	1	0
pRF148-1_119k_tetX	0	0	0	0	0	0	0	0	1	0
pRF52-1_119k_tetX	0	0	0	0	0	0	0	0	0	0
pRF108-2_97k_tetX	0	0	0	0	0	0	0	0	1	0
pRF45-1_31k_tetX	0	0	0	0	0	0	0	0	0	0
pRF14-1_50k_tetX	0	0	0	0	0	0	1	0	0	0
pRF10-1_119k_tetX	0	0	0	0	0	0	0	0	0	0
pRT18-1_294k_tetX	0	0	0	0	0	0	0	0	1	0
pRW7-1_235k_tetX	0	0	0	0	0	0	0	0	1	0
pRW8-1_122k_tetX	0	0	0	0	0	0	0	0	1	0
p2GS3-tetX4	0	0	0	0	0	0	0	0	0	0
pAB4-4-tetX4	0	0	0	0	0	0	0	0	1	0
pHN10R-tetX4	0	0	0	0	1	0	0	0	0	0

pHS2-1-tetX4	0	0	0	0	0	0	0	0	0	0
pHS10-1-tetX4	0	0	0	0	1	0	0	0	0	0
pHS19-2-tetX4	0	0	0	0	1	0	0	0	0	0
pSC4R-tetX4	0	0	0	0	0	0	0	0	0	0
pSDP9R-tetX4	0	0	0	0	0	0	0	0	0	0
pSH9W-tetX4	0	0	0	0	0	0	0	0	0	0
pSH12R-tetX4	0	0	0	0	0	0	0	0	0	0
pSX5G-tetX4	0	0	0	0	1	0	0	0	0	0
pSX8G-tetX4	0	0	0	0	0	0	0	0	1	0
pSZ5R-tetX4	0	0	0	0	0	0	0	0	0	0
pSZ6R-tetX4	0	0	0	0	0	0	0	0	1	0
pSZ10R-tetX4	0	0	0	0	0	0	0	0	1	0
pSZ11R-tetX4	0	0	0	0	1	0	0	0	0	0
pAB12-1-tetX4	0	0	0	0	0	0	0	0	0	0
pHN13R-tetX4	0	0	0	0	0	0	0	0	1	0

Plasmid	Antibiotic resistance gene									
	Aminoglycosides-resistance gene									
	aac(3)-IId	*aac(3)-IVa*	*aac(6')-Ib*	*aadA2*	*ant(3'')-Ia*	*aph(3')-Ia*	*aph(3'')-Ib*	*aph(3'')-Ib*	*aph(4)-Ia*	*aph(6)-Id*
pTECL_2-190k-tetX4	0	0	0	0	1	0	0	0	0	0
pLHM10-1	0	0	0	0	0	0	0	0	0	0
pYSP8-1	0	0	0	0	1	0	0	0	0	0
pG3X16-2-3	0	0	0	1	1	0	0	1	0	1
pYY76-1-2	0	0	0	1	0	0	0	0	0	0
p54-tetX	0	0	0	0	0	0	0	0	0	0
pYPE12-101k-tetX4	0	0	0	0	0	0	0	0	0	0
pYPE10-190k-tetX4	0	0	0	0	1	0	0	0	0	0
pYPE3-92k-tetX4	0	0	0	0	0	0	0	0	0	0
pNT1F31-tetX4	0	0	0	0	0	0	0	0	0	0
p1916D18-1	0	0	0	1	0	0	0	0	0	0
p1916D6-2	0	0	0	1	0	0	0	0	0	0
p1919D3-1	0	0	0	1	1	0	0	0	0	0
p1919D62-1	0	0	0	0	1	0	0	0	0	0
pT16R-1	0	0	0	0	1	0	0	0	0	0
pZF31-tetX-119kb	0	0	0	1	1	0	0	0	0	0
pZF34-tetX-114kb	0	0	0	1	1	0	0	0	0	0
p2EC1-1	0	1	0	0	0	0	0	0	1	0

p94EC-2	0	1	0	0	0	0	0	0	1	0
pEC931_tetX	0	0	0	1	0	0	0	0	0	0
pT28R-1	0	0	0	0	1	0	0	0	0	0
pCD58-3-1	0	0	0	0	0	0	0	0	0	1
pCD63-2-1	0	0	0	1	0	0	0	0	0	0
pCD74-2-2	0	0	0	0	0	0	0	0	0	1
pSTB20-1T	0	0	0	0	0	0	0	0	0	0
pHNCF11W-tetX4	0	0	0	1	0	0	0	0	0	0
pSY3626C1_315k	1	0	0	0	1	1	0	0	0	0
pSY3626_190k_tetX	0	0	0	0	1	0	0	0	0	0
pSal21GXH-tetX4	0	0	0	1	1	0	0	0	0	0
pPK5074-tetX	0	0	0	0	1	1	0	1	0	1
pNT1F10-tetX4	0	0	0	0	0	0	0	0	0	0
pNT1N34-tetX4	0	0	0	0	0	0	0	0	0	0
pNT1W22-tetX4	0	0	0	0	0	0	0	0	0	0
pNT1F25-tetX4	0	0	0	0	0	0	0	0	0	0
pNT1N28-tetX4	0	0	0	0	0	0	0	0	0	0
pNT1W25-tetX4	0	0	0	0	0	0	0	0	0	0
pNT1N31-tetX4	0	0	0	0	0	0	0	0	0	0
pNT1N25-tetX4	0	0	0	0	0	0	0	0	0	0
pNT1F34-tetX4	0	0	0	0	0	0	0	0	0	0
pNTT31XS-tetX4	0	0	0	0	0	0	0	0	0	0
pPK8217-tetX	0	0	0	0	1	1	0	1	0	1
pPK8568-tetX	0	0	0	0	0	0	0	0	0	0
pPK8277-tetX	0	0	0	0	0	0	0	0	0	0
pPK8241-tetX	0	0	0	0	0	0	0	0	0	0
pPK8276-tetX	0	0	0	0	0	0	0	0	0	0
pPK8261-tetX	0	0	0	0	0	0	0	0	0	0
pPK8275-tetX	0	0	0	0	0	0	0	0	0	0
pPK8566-tetX	0	0	0	0	0	0	0	0	0	0
pPK5086-tetX	0	0	0	0	0	0	0	0	0	0
pNUITM-VK5_mdr	1	0	1	0	1	0	0	1	0	1
p47EC	0	0	0	1	0	1	0	0	0	0
p2019XSD11-190	0	0	0	0	1	0	0	0	0	0
p16EC-9K	0	0	0	0	0	0	0	0	0	0
pEC05-X4	0	0	0	1	0	0	0	0	0	0
pEC12-X4	0	0	0	1	0	0	0	0	0	0
pRB3-1_31K_tetX	0	0	0	1	0	0	0	0	0	0

pRF173-1_87k_tetX	0	0	0	0	0	0	0	0	0	0
pRF148-2_101k_tetX	0	0	0	0	0	0	0	0	0	0
pRF148-1_119k_tetX	0	0	0	1	1	0	1	0	0	1
pRF52-1_119k_tetX	0	0	0	1	1	0	1	0	0	1
pRF108-2_97k_tetX	0	0	0	0	0	0	0	0	0	0
pRF45-1_31k_tetX	0	0	0	1	0	0	0	0	0	0
pRF14-1_50k_tetX	0	0	0	0	0	1	0	0	0	0
pRF10-1_119k_tetX	0	0	0	1	1	0	1	0	0	1
pRT18-1_294k_tetX	0	0	0	0	1	0	0	0	0	0
pRW7-1_235k_tetX	0	0	0	0	1	1	0	0	0	0
pRW8-1_122k_tetX	0	0	0	1	1	0	0	0	0	1
p2GS3-tetX4	0	0	0	0	0	0	0	0	0	0
pAB4-4-tetX4	0	0	0	0	1	0	0	0	0	0
pHN10R-tetX4	0	0	0	1	0	0	0	0	0	0
pHS2-1-tetX4	0	0	0	0	0	0	0	0	0	0
pHS10-1-tetX4	0	0	0	1	0	0	0	0	0	0
pHS19-2-tetX4	0	0	0	1	0	0	0	0	0	0
pSC4R-tetX4	0	0	0	0	0	1	0	0	0	0
pSDP9R-tetX4	0	0	0	0	0	0	0	0	0	0
pSH9W-tetX4	0	0	0	1	0	0	0	0	0	0
pSH12R-tetX4	0	0	0	0	0	0	0	0	0	0
pSX5G-tetX4	0	0	0	1	0	0	0	0	0	0
pSX8G-tetX4	0	0	0	0	1	0	0	0	0	0
pSZ5R-tetX4	0	0	0	1	0	0	0	0	0	0
pSZ6R-tetX4	0	0	0	0	1	0	0	0	0	0
pSZ10R-tetX4	0	0	0	1	1	0	0	1	0	1
pSZ11R-tetX4	0	0	0	1	0	0	0	0	0	0
pAB12-1-tetX4	1	0	0	0	0	0	0	0	0	0
pHN13R-tetX4	0	0	0	0	1	0	0	0	0	0

Plasmid	Antibiotic resistance gene							
	Sulfonamides-resistance gene						Lincomycin-resistance gene	
	dfrA5	*dfrA12*	*dfrA14*	*dfrA17*	*sul2*	*sul3*	*lnu*(F)	*lnu*(G)
pTECL_2-190k-tetX4	0	0	0	0	0	0	0	1
pLHM10-1	0	0	0	0	0	0	0	0
pYSP8-1	0	0	0	0	0	0	0	1
pG3X16-2-3	0	1	0	0	0	1	0	0
pYY76-1-2	0	0	0	0	0	0	1	0

p54-tetX	1	0	0	0	0	1	0	0
pYPE12-101k-tetX4	1	0	0	0	0	1	0	0
pYPE10-190k-tetX4	0	0	0	0	0	0	0	1
pYPE3-92k-tetX4	1	0	0	0	0	1	0	0
pNT1F31-tetX4	1	0	0	0	0	1	0	0
p1916D18-1	0	0	0	0	0	0	0	1
p1916D6-2	0	0	0	0	0	0	1	0
p1919D3-1	0	1	0	0	0	1	0	1
p1919D62-1	0	0	0	0	0	0	0	1
pT16R-1	0	0	0	0	0	0	0	1
pZF31-tetX-119kb	0	1	0	0	1	1	0	0
pZF34-tetX-114kb	0	1	0	0	1	1	0	0
p2EC1-1	0	0	0	0	0	0	0	0
p94EC-2	0	0	0	0	0	0	0	0
pEC931_tetX	0	0	0	0	0	0	1	0
pT28R-1	0	0	0	0	0	0	0	1
pCD58-3-1	0	0	1	0	0	1	0	0
pCD63-2-1	0	1	0	0	1	1	0	0
pCD74-2-2	0	0	1	0	0	1	0	0
pSTB20-1T	1	0	0	0	0	1	0	0
pHNCF11W-tetX4	0	0	0	0	0	0	1	0
pSY3626C1_315k	0	0	0	0	1	1	1	1
pSY3626_190k_tetX	0	0	0	0	0	0	0	1
pSal21GXH-tetX4	0	1	0	0	1	1	0	0
pPK5074-tetX	0	0	0	0	1	0	0	0
pNT1F10-tetX4	1	0	0	0	0	1	0	0
pNT1N34-tetX4	1	0	0	0	0	1	0	0
pNT1W22-tetX4	1	0	0	0	0	1	0	0
pNT1F25-tetX4	1	0	0	0	0	1	0	0
pNT1N28-tetX4	1	0	0	0	0	1	0	0
pNT1W25-tetX4	1	0	0	0	0	1	0	0
pNT1N31-tetX4	1	0	0	0	0	1	0	0
pNT1N25-tetX4	1	0	0	0	0	1	0	0
pNT1F34-tetX4	1	0	0	0	0	1	0	0
pNTT31XS-tetX4	0	0	0	0	0	0	0	0
pPK8217-tetX	0	0	0	0	1	0	0	0
pPK8568-tetX	0	0	0	0	0	0	0	0
pPK8277-tetX	0	1	0	0	0	0	0	0
pPK8241-tetX	0	0	0	0	0	0	0	0

pPK8276-tetX	0	0	0	0	0	0	0	0
pPK8261-tetX	0	1	0	0	0	0	0	0
pPK8275-tetX	0	1	0	0	0	0	0	0
pPK8566-tetX	0	1	0	0	0	0	0	0
pPK5086-tetX	0	1	0	0	0	0	0	0
pNUITM-VK5_mdr	0	0	0	1	1	0	0	0
p47EC	0	0	0	0	0	1	1	0
p2019XSD11-190	0	0	0	0	0	0	0	1
p16EC-9K	0	0	0	0	0	0	0	0
pEC05-X4	0	0	0	0	0	0	1	0
pEC12-X4	0	0	0	0	0	0	1	0
pRB3-1_31K_tetX	0	0	0	0	0	0	1	0
pRF173-1_87k_tetX	0	0	0	0	0	0	0	0
pRF148-2_101k_tetX	1	0	0	0	0	1	0	0
pRF148-1_119k_tetX	0	1	0	0	0	1	0	0
pRF52-1_119k_tetX	0	1	0	0	0	1	0	0
pRF108-2_97k_tetX	1	0	0	0	0	1	0	0
pRF45-1_31k_tetX	0	0	0	0	0	0	1	0
pRF14-1_50k_tetX	0	0	0	0	0	0	0	0
pRF10-1_119k_tetX	0	1	0	0	0	1	0	0
pRT18-1_294k_tetX	0	0	0	0	0	0	0	1
pRW7-1_235k_tetX	0	0	0	0	0	0	0	1
pRW8-1_122k_tetX	0	1	0	0	0	1	0	0
p2GS3-tetX4	0	0	0	0	1	0	0	0
pAB4-4-tetX4	0	0	0	0	0	0	0	1
pHN10R-tetX4	0	0	0	0	0	0	1	0
pHS2-1-tetX4	0	0	0	0	0	0	0	0
pHS10-1-tetX4	0	0	0	0	0	0	1	0
pHS19-2-tetX4	0	0	0	0	0	0	1	0
pSC4R-tetX4	0	0	0	0	0	0	0	0
pSDP9R-tetX4	0	0	0	0	0	0	0	0
pSH9W-tetX4	0	0	0	0	0	0	1	0
pSH12R-tetX4	0	0	0	0	0	0	0	0
pSX5G-tetX4	0	0	0	0	0	0	1	0
pSX8G-tetX4	0	0	0	0	0	0	0	1
pSZ5R-tetX4	0	0	0	0	0	0	1	0
pSZ6R-tetX4	0	0	0	0	0	0	0	1
pSZ10R-tetX4	0	1	0	0	0	1	0	0
pSZ11R-tetX4	0	0	0	0	0	0	1	0

pAB12-1-tetX4	0	0	0	0	1	0	0	0
pHN13R-tetX4	0	0	0	0	1	0	0	0

Plasmid	Antibiotic resistance gene				
	Macrolides-resistance gene				Fosfomycin-resistance gene
	erm(42)	*erm*(B)	*mph*(A)	*mef*(B)	*fosA3*
pTECL_2-190k-tetX4	0	0	0	0	0
pLHM10-1	0	0	0	0	0
pYSP8-1	0	1	0	0	0
pG3X16-2-3	1	0	0	0	0
pYY76-1-2	0	0	0	0	0
p54-tetX	0	0	0	1	0
pYPE12-101k-tetX4	0	0	0	1	0
pYPE10-190k-tetX4	0	0	0	0	0
pYPE3-92k-tetX4	0	0	0	1	0
pNT1F31-tetX4	0	0	0	1	0
p1916D18-1	0	0	0	0	0
p1916D6-2	0	0	0	0	0
p1919D3-1	0	0	0	0	0
p1919D62-1	0	0	0	0	0
pT16R-1	0	0	0	0	0
pZF31-tetX-119kb	1	0	0	0	0
pZF34-tetX-114kb	1	0	0	0	0
p2EC1-1	0	0	0	0	0
p94EC-2	0	0	0	0	1
pEC931_tetX	0	0	0	0	0
pT28R-1	0	0	0	0	0
pCD58-3-1	0	0	0	0	0
pCD63-2-1	0	0	0	0	0
pCD74-2-2	0	0	0	0	0
pSTB20-1T	0	0	0	1	0
pHNCF11W-tetX4	0	0	0	0	0
pSY3626C1_315k	0	0	0	0	0
pSY3626_190k_tetX	0	0	0	0	0
pSal21GXH-tetX4	0	0	0	0	0
pPK5074-tetX	0	0	0	0	0
pNT1F10-tetX4	0	0	0	1	0
pNT1N34-tetX4	0	0	0	1	0
pNT1W22-tetX4	0	0	0	1	0
pNT1F25-tetX4	0	0	0	1	0
pNT1N28-tetX4	0	0	0	1	0
pNT1W25-tetX4	0	0	0	1	0
pNT1N31-tetX4	0	0	0	1	0
pNT1N25-tetX4	0	0	0	1	0

pNT1F34-tetX4	0	0	0	1	0
pNTT31XS-tetX4	0	0	0	0	0
pPK8217-tetX	0	0	0	0	0
pPK8568-tetX	0	0	0	0	0
pPK8277-tetX	0	0	1	0	1
pPK8241-tetX	0	0	0	0	0
pPK8276-tetX	0	0	0	0	0
pPK8261-tetX	0	0	1	0	1
pPK8275-tetX	0	0	1	0	1
pPK8566-tetX	0	0	1	0	1
pPK5086-tetX	0	0	1	0	1
pNUITM-VK5_mdr	0	0	0	0	0
p47EC	1	0	0	0	0
p2019XSD11-190	0	0	0	0	0
p16EC-9K	0	0	0	0	0
pEC05-X4	0	0	0	0	0
pEC12-X4	0	0	0	0	0
pRB3-1_31K_tetX	0	0	0	0	0
pRF173-1_87k_tetX	1	0	0	0	0
pRF148-2_101k_tetX	0	0	0	1	0
pRF148-1_119k_tetX	0	0	1	0	0
pRF52-1_119k_tetX	1	0	0	0	0
pRF108-2_97k_tetX	0	0	0	1	0
pRF45-1_31k_tetX	0	0	0	0	0
pRF14-1_50k_tetX	0	0	0	0	0
pRF10-1_119k_tetX	1	0	0	0	0
pRT18-1_294k_tetX	0	0	0	0	0
pRW7-1_235k_tetX	0	0	0	0	0
pRW8-1_122k_tetX	0	0	1	0	0
p2GS3-tetX4	0	0	0	0	0
pAB4-4-tetX4	0	0	0	0	0
pHN10R-tetX4	0	0	0	0	0
pHS2-1-tetX4	0	0	0	0	0
pHS10-1-tetX4	0	0	0	0	0
pHS19-2-tetX4	0	0	0	0	0
pSC4R-tetX4	0	0	0	0	0
pSDP9R-tetX4	0	0	0	0	0
pSH9W-tetX4	0	0	0	0	0
pSH12R-tetX4	0	0	0	0	0
pSX5G-tetX4	0	0	0	0	0
pSX8G-tetX4	0	0	0	0	0
pSZ5R-tetX4	0	0	0	0	0
pSZ6R-tetX4	0	0	0	0	0
pSZ10R-tetX4	1	0	1	0	0

pSZ11R-tetX4	0	0	0	0	0
pAB12-1-tetX4	1	0	0	0	0
pHN13R-tetX4	1	0	0	0	0
